# Clinical Presentation of Celiac Disease and Diagnosis Accuracy in a Single-Center European Pediatric Cohort over 10 Years

**DOI:** 10.3390/nu13114131

**Published:** 2021-11-18

**Authors:** Anna Rita Di Biase, Giovanni Marasco, Federico Ravaioli, Luigi Colecchia, Elton Dajti, Marco Lecis, Erica Passini, Luigina Vanessa Alemanni, Davide Festi, Lorenzo Iughetti, Antonio Colecchia

**Affiliations:** 1 Pediatric Unit, Modena University Hospital, 41124 Modena, Italy; colecchiadibi@libero.it (A.R.D.B.); marcolecis@hotmail.it (M.L.); erica.passini11@gmail.com (E.P.); lorenzo.iughetti@unimore.it (L.I.); 2 Department of Digestive Diseases, IRCCS Azienda Ospedaliero-Universitaria di Bologna, 40126 Bologna, Italy; f.ravaioli@unibo.it (F.R.); e_dajti17@hotmail.com (E.D.); vanessaalemanni1@gmail.com (L.V.A.); 3 Department of Medical and Surgical Sciences, University of Bologna, 40126 Bologna, Italy; luigi.colecchia@studio.unibo.it (L.C.); davide.festi@unibo.it (D.F.); 4 Gastroenterology Unit, Modena University Hospital, 41124 Modena, Italy; antonio.colecchia@aovr.veneto.it

**Keywords:** celiac disease, ESPGHAN, transglutaminase, esophagogastroduodenoscopy, pediatric age

## Abstract

(1) Background: Changes in the clinical presentation of celiac disease (CD) in children have been reported. The guidelines of the European Society of Pediatric Gastroenterology, Hepatology and Nutrition (ESPGHAN) allow esophagogastroduodenoscopy (EGD) with biopsies to be avoided under specific circumstances. We aimed to assess the clinical picture of pediatric CD patients at diagnosis and to validate ESPGHAN non-biopsy criteria. (2) Methods: Patients with suspected CD or undergoing screening from 2004 to 2014 at the University Hospital in Modena, Italy were enrolled. The accuracy of ESPGHAN non-biopsy criteria and modified versions were assessed. (3) Results: In total, 410 patients were enrolled, of whom 403 were considered for analysis. Of the patients considered, 45 were asymptomatic and diagnosed with CD (11.2%) while 358 patients (88.2%) were symptomatic, of whom 295 were diagnosed with CD. Among symptomatic CD patients, 57 (19.3%) had gastrointestinal symptoms, 98 (33%) had atypical symptoms and 140 (47.4%) had both. No difference was found for the presence of gastrointestinal symptoms at different ages. The non-biopsy ESPGHAN criteria yielded an accuracy of 59.4% with a positive predictive value (PPV) of 100%; 173 out of 308 EGD (56.2%) could have been avoided. The modified 7× and 5× upper limit of normal cut-offs for IgA anti tissue-transglutaminase reached 60.7% and 64.3% of EGD avoided, respectively. (4) Conclusions: Over 10 years, late age at diagnosis and increased rates of atypical CD presentation were found. ESPGHAN non-biopsy criteria are accurate for CD diagnosis and allow half of unneeded EGD to be avoided. Modified versions allowed sparing a greater number of EGD.

## 1. Introduction

Celiac disease (CD) is a chronic small intestinal immune-mediated enteropathy triggered and maintained by dietary gluten in genetically predisposed individuals [1]. In recent decades, the disease incidence has increased significantly [2,3,4,5], constituting a major health problem affecting up to 1–3% of children [6,7]. Concurrent with the increasing incidence, changes in the clinical presentation have been observed since the 1980s [8]. Moreover, differences in CD prevalence and presentation between closely located geographic areas and fluctuations within the same country have been reported [9,10]. Furthermore, the average age at diagnosis has risen from <2 to 6–9 years in many developed countries [2,3,11,12], mainly due to the increasing number of asymptomatic patients diagnosed with CD. The main reasons underlying the incidence trend of CD [3] may be due to an increased awareness among physicians, the screening of at-risk groups [13] and the adoption of new serologic tools [2,11]. Indeed, while the small intestinal biopsy was considered the gold standard for CD diagnosis in Europe according to the guidelines released by the European Society for Pediatric Gastroenterology, Hepatology and Nutrition (ESPGHAN) since 1969 [14], the revision carried out in 2012 stated that biopsy could be omitted under specific circumstances [1]. Symptomatic children with serum anti-tissue transglutaminase (anti-tTG) antibody levels ≥ 10 times the upper limit of normal (ULN) can avoid duodenal biopsies after positive human leukocyte antigen (HLA) testing and serum anti-endomysial antibodies (EMAs) positivity [1] with a good accuracy. Indeed, the reported sensitivity and specificity for the anti-tTG test was 96% and 99%, and 95% and 100% for EMA, respectively [1,15,16]. Several authors tried to validate these criteria in specific geographical areas with different CD prevalence and clinical presentation [17]. However, data regarding the clinical presentation of CD in the last decade and the validation of new ESPGHAN criteria for the non-invasive diagnosis of CD from a large Italian pediatric cohort are still scarce. Thus, we aimed to characterize trends in the clinical presentation of CD in a large pediatric cohort from Northern Italy diagnosed over 10 years and to evaluate the diagnostic performance of new ESPGHAN criteria [1] for the non-invasive diagnosis of CD.

## 2. Materials and Methods

Data of consecutive patients referred to the Pediatric Gastroenterology Unit of the Department of Pediatrics of the Modena University Hospital in Modena, Italy were collected. We included all patients aged ≤18 years referred for CD clinical suspicion or screening during the study period from 1 January 2004 until 31 December 2014. The study was conducted following the Helsinki Declaration and approved by the local Ethical Committee.

### 2.1. Demographics and Clinical Data

The following demographic characteristics from the patients’ medical records were collected: age, gender, age at first symptoms presentation, year of symptoms presentation, height and weight, body mass index, date of diagnosis of CD and family history for CD. Data on the presence and kind of symptoms at presentation were also collected. In particular, according to ESPGHAN guidelines [1], patients with diarrhea, weight loss, failure to thrive, anorexia, abdominal distention, abdominal pain, short stature, flatulence, irritability, increased titers of liver enzymes, constipation and anemia were considered as symptomatic for CD. On the other hand, asymptomatic children were those without any symptoms receiving screening for CD if they were first-degree relatives of CD patients or included in at-risk groups (i.e., other auto-immune diseases, etc.). To obtain reliable results, we standardized subjective symptoms according to the following definition for chronic diarrhea (defecation ≥ 3 times a day during at least 14 days) [18], failure to thrive (deflective weight-to-height curve or being ≤−2 standard deviations (SDs) off on the weight-to-length curve), short stature (≥2 SDs smaller than children with the same age, or as being ≥2 SDs under their target height) [19], anemia (based on hematological references for children) [20], recurrent abdominal pain (intermitting abdominal pain for ≥2 months) [21], constipation (defecation ≤ 3 times a week or with pain during defecation with the production of hard stools) [22] and bloating (recurrent sensation of abdominal distention that may or may be not associated with measurable distention) [23]. When the definitions were not applicable to the available information, we searched for the description of the symptoms in the physician’s evaluation paper.

### 2.2. Serological Tests and EGD Biopsy

The results of the different serological tests for CD (IgA levels, anti-tTG IgA, EMA, HLA haplotypes) were collected; anti-tTG IgA levels and EMA and serum IgA levels were measured. When IgA deficiency (defined as <0.20 g/L) was present, IgG levels of anti-tTG were measured. An anti-tTG level > 10 U/mL was considered positive. IgA anti-tTG antibodies were assayed on commercially available ELISA kits (cut-off value > 10 UA/mL for anti-tTG). We also collected data on biopsy samples taken during EGD; histological lesions were graded according to the Marsh criteria (0 = normal mucosa; 1 = increased number of intraepithelial lymphocytes; 2 = crypt hyperplasia; 3 = villous atrophy) [1].

### 2.3. CD Diagnosis

Positive CD cases were diagnosed until the end of 2011 according to the old ESPGHAN guidelines [14] and in 2012 according to the new ESPGHAN guidelines [1]. According to the old guidelines [14], every CD serology (anti-tTG/EMA)-positive patient had to be confirmed histologically by an EGD with duodenal biopsy. The presence of a Marsh–Oberhuber grade ≥ 2 was diagnostic for CD. On the other hand, according to the new 2012 ESPGHAN guidelines, children with symptoms with an anti-tTG titer > 10× ULN, a positive EMA and positive HLA haplotype (DQ2 or DQ8) were diagnosed for CD and thus, EGD was avoidable. In all other cases, an EGD with a duodenal biopsy was necessary. In these children, a Marsh grade ≥ 2 allowed CD diagnosis.

### 2.4. Statistical Analysis

Continuous variables were reported as mean and standard deviations (SD), while categorical variables were reported as number and percentage. Variables were compared using the Student *T*-test, Mann–Whitney U test, Chi^2^ or Fisher’s exact tests when appropriate. Trends in the presence of symptoms at CD diagnosis between the period 2004–2009 and 2009–2014 and at different ages (toddler age (0–3 years), primary school age (4–12 years) and high school age (>12 years)) were evaluated. The accuracy, sensitivity, specificity and positive and negative predictive values (PPV and NPV) with their 95% confidence intervals (95% CI) of the new ESPGHAN 2012 criteria for CD diagnosis on the group of symptomatic patients diagnosed with CD with duodenal biopsies until 2011 were evaluated. Finally, the number of EGD that could have been avoided using the new ESPGHAN 2012 criteria (IgA anti-tTG ≥ 10 times ULN) was obtained. Since different cut-offs for IgA anti-tTG have been previously proposed, we made the same evaluation using previously proposed cut-offs (5× and 7× ULN). The probability values are two-sided; a probability value (*p*) less than 0.05 was considered statistically significant. Statistical analysis was performed with STATA 13.0 (College Station, TX, USA: StataCorp LP).

## 3. Results

### 3.1. Demographics and Clinical Characteristics

**Figure 1:**
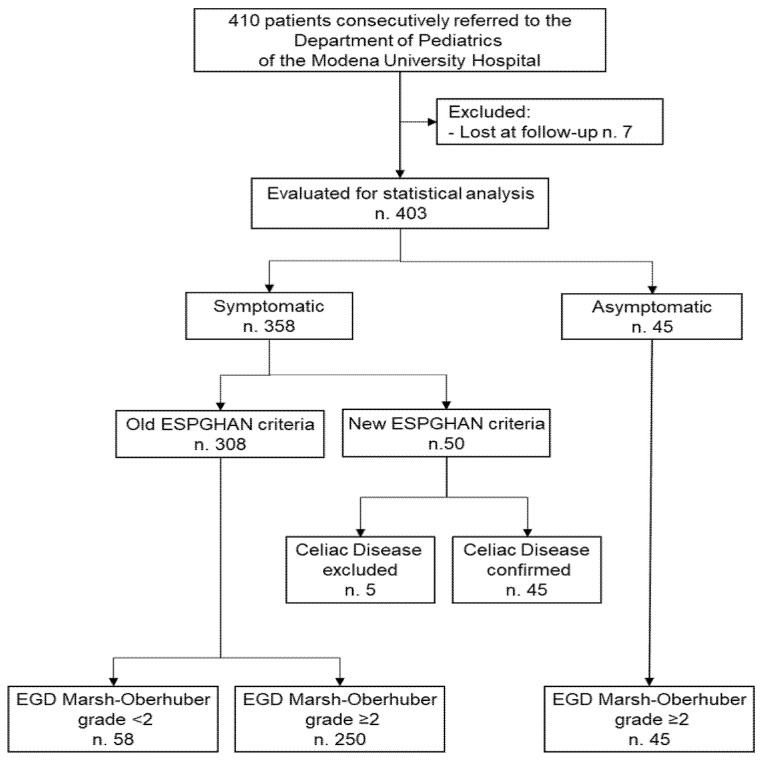
Flow-chart of the selection of patients enrolled in the study.

**Figure 2:**
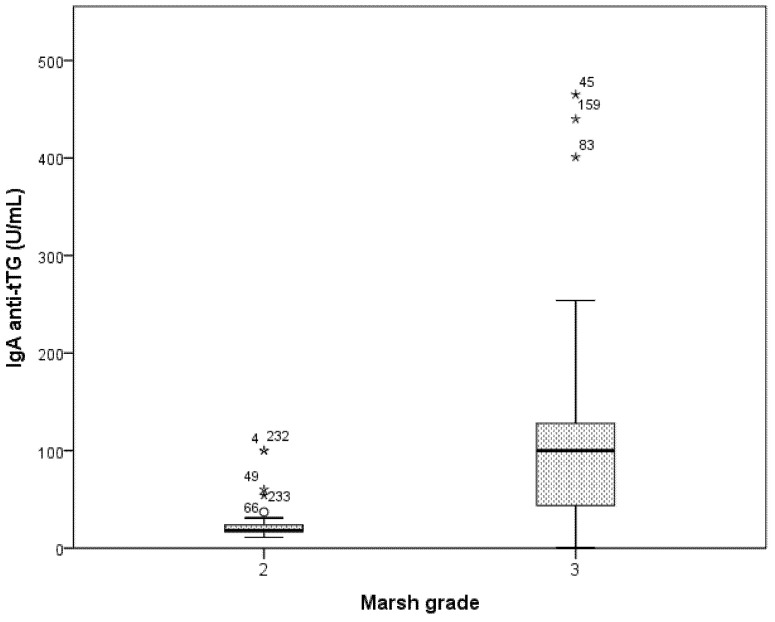
Anti-tissue transglutaminase IgA antibodies levels according to Marsh grade. Abbreviations: IgA, immunoglobulin A; tTG, tissue transglutaminase; UI, International Units; mL, milliliters. * outliers.

During the study period, of the 410 patients consecutively referred to the Department of Pediatrics of the Modena University Hospital, 7 patients (1.7%) were excluded for incomplete data. Among the remaining 403 patients, 45 (11.2%) were asymptomatic and were evaluated because they were at high risk for CD; all these 45 patients were diagnosed with CD. The remaining 358 patients (88.2%) were evaluated for the presence of symptoms suggestive of CD and all of them were diagnosed with CD except for 63 (17.6%) patients (58 diagnosed with old ESPGHAN criteria and 5 diagnosed with new 2012 ESPGHAN criteria). Among the 295 symptomatic patients with CD, in all patients the diagnosis was made with old ESPGHAN criteria except for 45 patients diagnosed according to the new ESPGHAN criteria from January 2012. Figure 1 reports the detailed enrollment flow chart. The mean age at CD diagnosis was 76.6 months (SD 44.3). Most of the patients enrolled were female (223 out of 340 CD patients, 65.6%). The mean BMI percentile at diagnosis was 38.1 (SD 30.4). Anti-tTG antibody levels were correlated to Marsh grade (Marsh 2 mean 25.4 U/mL (SD 19.7) vs. Marsh 3 mean 91.2 U/mL (SD 62.6), *p* < 0.001) (Figure 2). Other demographics and clinical characteristics of patients enrolled are reported in Table 1.

### 3.2. Clinical Presentation

Among the 295 symptomatic patients with CD, 57 (19.3%) reported gastrointestinal symptoms, 98 (33%) reported atypical extra-intestinal symptoms and 140 (47.4%) reported both, corresponding to a cumulative prevalence of gastrointestinal and extra-intestinal symptoms of 66.8% and 80.7%, respectively. The presence of symptoms in CD patients was higher in the period 2004–2009 compared to 2009–2014, although without statistical significance (47 (46.5%) vs. 75 (36.6%), respectively, *p* = 0.095).

Stratifying for symptoms and associated conditions, we found that asthenia was the only factor more prevalent in the period 2009–2014 (11.2% vs. 3.1%, *p* = 0.018) (Table 2).

No difference was found for the presence of overall gastrointestinal symptoms at different ages (52.8% vs. 37.5% vs. 30% for toddler, primary and high school age, respectively, *p* = 0.086). Abdominal pain was more prevalent in high and primary school age compared to toddler age (52% vs. 39.5% vs. 18.6%, respectively, *p* = 0.001). Bloating, slow growth, weight loss and high transaminase levels were more prevalent in toddler age than primary and high school age (*p* < 0.05). On the contrary, in school-age patients, headache, asthenia and their simultaneous presence were more reported (*p* ≤ 0.001) (Table 3).

### 3.3. Accuracy of ESPGHAN Criteria in Symptomatic Patients

A total of 308 patients evaluated for the presence of symptoms with old ESPGHAN criteria were considered; among 58 patients without CD, 10 patients had positive anti-tTG IgA, but none had a title of anti-tTG IgA higher than 10× ULN. On the other hand, among the remaining 250 patients diagnosed for CD with EGD and biopsy, 125 had an anti-tTG IgA title higher 10× ULN (true positive). Thus, the new ESPGHAN criteria for sparing EGD in symptomatic patients in our enrolled population reached an overall accuracy of 59.4% (95% CI 53.7–65), with a specificity of 100.0% (95% CI 93.8–100), sensitivity of 50% (95% CI 43.6–56.4), PPV of 100% and NPV of 31.7% (95% CI 29.1–34.4). Accordingly, 173 out of 308 EGD (56.2%) could have been avoided in the past with the new ESPGHAN criteria for sparing EGD in symptomatic patients, with a CD missing rate of 0%.

### 3.4. Accuracy of Modified ESPGHAN Criteria in Symptomatic Patients

Among the 58 patients with symptoms but without CD, only 1 patient had a title of anti-tTG higher than 7× ULN. Among the remaining 250 patients diagnosed for CD, 138 had an anti-tTG IgA title higher than 7× ULN (true positive). Thus, the modified 7× ULN cut-off for sparing EGD reached an overall accuracy of 63.3% (95% CI 57.7–68.7), with a specificity of 98.3% (95% CI 90.8–99.7), sensitivity of 55.2% (95% CI 48.8–61.5), PPV of 99.3% (95% 95.2–99.9) and NPV of 33.7% (95% CI 30.6–37). Accordingly, a greater number of unneeded EGD (187 out of 308 EGD, 60.7%) could have been avoided when compared to the new ESPGHAN criteria (*p* = 0.258), with a negligible CD missing rate of 0.3% (*p* = 0.336).

**Figure 3:**
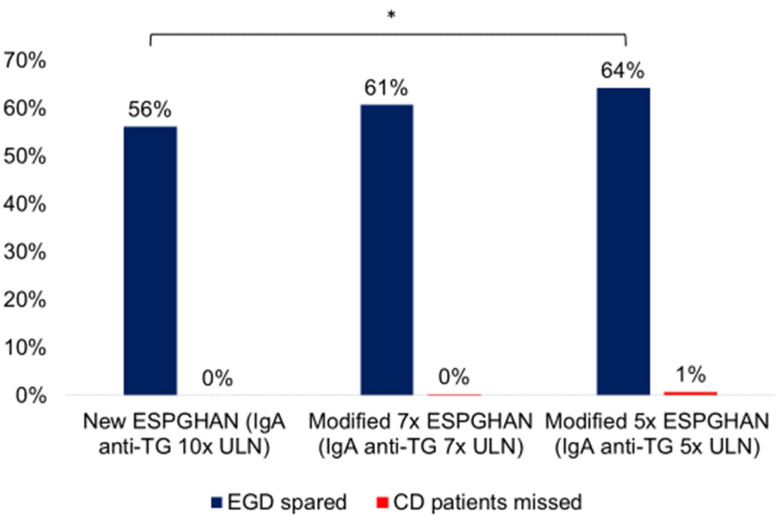
Performance of new and modified (7× and 5×) ESPGHAN criteria in symptomatic CD patients (* for statistically significant differences, *p* < 0.05). Abbreviations: IgA, immunoglobulin A; tTG, tissue transglutaminase; ULN, upper limit of normal; EGD, esophagogastroduodenoscopy; CD, celiac disease.

When 5× ULN cut-off was considered, among the 58 patients without CD, only 2 patients had a title of anti-tTG higher than 5× ULN. Among the remaining 250 patients diagnosed with CD, 148 had an anti-tTG IgA title higher than 5× ULN (true positive). Thus, the modified 5× ULN cut-off for sparing EGD reached an overall accuracy of 66.2% (95% CI 60.7–71.5), with a specificity of 96.6% (95% CI 88.1–99.6), sensitivity of 59.2% (95% CI 52.8–65.4), PPV of 98.7% (95% 95–99.7) and NPV of 35.4% (95% CI 31.9–39.1). Accordingly, a significant number of unneeded EGD (198 out of 308, 64.3%) could have been avoided when compared to the new ESPGHAN criteria (*p* = 0.040), with a negligible CD missing rate of 0.8% (*p* = 0.116). Figure 3 shows the performance of new and modified ESPGHAN criteria.

## 4. Discussion

Our study aimed to describe the clinical presentation of pediatric CD over 10 years in a tertiary referral pediatric unit. Most of the patients included were children (4–12 years old) and symptomatic. No trend in symptom presentation was found between two consecutive five-year periods. Interestingly, abdominal pain, headache, and asthenia were more prevalent in children and teenagers, whereas bloating, slow growth, weight loss and high transaminase levels were more prevalent in toddlers. Basing on a biopsy-proven CD population, we showed that the ESPGHAN non-biopsy criteria allow a correct diagnosis of CD. The efficacy of these criteria was confirmed even when modified cut-offs of IgA anti-tTG antibodies were employed, since the PPV remained higher than 95% and the CD missing rate was <1%. Moreover, more than 50% of EGD performed in children may have been safely avoided, thus reducing the burden of such invasive examination and its cost for health care. Different IgA anti-tTG cut-offs may allow sparing a greater number of EGD while maintaining appropriate accuracy.

Changes in the demographics of CD presentation and clinical pattern have been observed since the 1980s [8]. In the present study, we confirmed previous observations of an increased prevalence of CD in females [24,25], accounting for more than 60% in our unselected cohort. Moreover, about 13% of our cohort was diagnosed after screening for CD family history or other autoimmune diseases. The rate of asymptomatic CD patients in our study perfectly traces that reported in the 2000s by a Swedish study assessing demographics and clinical evolution of CD over the period 1973–2013 [26]. However, these data are in contrast with the results of a previous study from Spain which reported only 7% of asymptomatic patients [27]. This discrepancy may underline the effect of different genetic, environmental and nutritional factors on CD clinical presentation [28,29,30,31]; further causes of divergence can be the local screening policies leading to CD diagnosis in early stages.

In addition, most of our patients were diagnosed with CD at child or toddler age, which differs from historical data reporting diagnosis at <2 years on average [8]. However, the median age of our cohort’s diagnosis was about 6 years, in line with several other reports [2,3,11,12]. This increased age at CD diagnosis may be due to different feeding practices influencing clinical presentation and the age of diagnosis. Similar to previous studies from Europe [15,16,32] and the U.S.A. [33], we showed that the clinical spectrum of CD shifted to an increase in atypical presentation instead of classical symptoms. Among the symptoms reported, abdominal pain, bloating, slow growth and anemia were the most frequent. Nevertheless, significant differences were also found for other atypical symptoms such as headache and asthenia, which were more prevalent in high-school-age children, confirming previous data from northern Europe [26]. However, the rate of symptom presentation at different ages can be influenced both by the age-related ability of the patient to describe the symptoms and by the parents’ assessment, which may also overestimate the clinical picture. Notably, to partially overcome this limit and standardize clinical manifestations, we used objective definitions to describe symptoms. We reported a prevalence of gastrointestinal symptoms of 70%, in line with previous experiences [26,34]. Interestingly, instead of chronic diarrhea, abdominal pain was the most prevalent symptom of our cohort, similar to a previous Dutch study [15]. Moreover, as part of changing the CD clinical scenario, slow growth was reported in only 18.3% of our cohort, in contrast to other studies [26,34]. Similarly, the greater prevalence of anemia reported by the present study when compared to others [35] may be due to the more intensive screening and awareness of CD.

Our study also showed an excellent performance of ESPGHAN non-biopsy criteria. Moreover, we found that the specificity of IgA anti-TG was 98.3% and 96.6% for 7× and 5× ULN, respectively, in line with a previous meta-analysis (range 77.8–100%) [36]. A recent study from the Mediterranean area including Italy showed that the anti-tTG 10× ULN PPV was 96.1%, in line with our data [37]. Similar results on anti-tTG 10× ULN PPV have also been reported in a cohort from New Zealand [38], suggesting that 50–60% of patients could avoid EGD and biopsy by applying the new ESPGHAN criteria [38]. Moreover, another multicentric study including 412 children from Central Europe reported that 48.4% of patients could avoid EGD [39]. Lower rates of applicability of the new ESPGHAN criteria have been reported in southern Europe (154/749, 20.7%), mainly due to the unavailability of HLA testing [40]. On the contrary, our results are in line with those of Werkstetter et al. [41], where 399 out of 707 (56.4%) patients were found with an anti-tTG title > 10× ULN. Moreover, in line with previous studies not including healthy subjects [42,43], we found that the sensitivity of the new ESPGHAN criteria was rather low, namely 50% in our cohort, suggesting that these criteria are adequate only for ruling-out rather than ruling-in CD diagnosis.

While serology’s accuracy and predictive value for the non-invasive assessment of pediatric CD has been extensively reported, only few studies have focused on the number of EGD that could have been avoided [38,39,44]. In our cohort, 56.2% of EGD could have been avoided with a CD missing rate of 0%. The advantage of avoiding invasive tests as EGD could allow general anesthesia or deep sedation to be eliminated, which are required to perform EGD in many centers, cancelling out its related risks and decreasing the costs [45]. For this purpose, lower cut-offs of anti-tTG IgA for symptomatic patients have been previously proposed. Gidrewicz et al. [44] reported that titers of anti-tTG IgA 3–10× ULN led to sparing up to 49% of unneeded EGD. We also observed that lower cut-offs of anti-tTG IgA 5–7× ULN could safely spare EGD in 60.7% and 64.3% of patients, respectively. Nevertheless, according to the recent update of non-biopsy ESPGHAN criteria [17], due to inter-laboratory variability, the employment of anti-tTG IgA levels < 10× ULN would not be recommended since they are more prone to technical error and are insufficient for avoiding EGDs and biopsy. However, in their systematic review of the available literature, even anti-tTG IgA cut-offs from 5× to 7.5× ULN still yielded optimal PPV values, ranging from 92.3% to 100%. Our study has some limits. Being a tertiary pediatric center, only patients with high CD suspicion were referred; this may have influenced the high CD prevalence found in our cohort, biasing the PPV of anti-tTG, which is dependent on disease prevalence [46]. However, other large studies from tertiary centers have reported high prevalence of CD [41]. Another limiting factor is the retrospective design, which was partially overcome by the consecutive enrollment in a pediatric gastroenterology outpatient facility, thus minimizing the risk of a selection bias.

## 5. Conclusions

In conclusion, no trend in symptoms presentation was found between two consecutive five-years period. Over 10 years, we found increased age at CD diagnosis and higher rates of atypical disease presentation. Finally, ESPGHAN non-biopsy criteria allowed a correct diagnosis of CD, avoiding more than half of unneeded EGD. The employment of lower antibody cut-offs for non-biopsy criteria led to sparing a greater number of EGDs. Further prospective studies should standardize laboratory testing for anti-tissue transglutaminase antibodies in order to safely apply these new cut-offs.

## Figures and Tables

**Table 1:** Demographic and clinical characteristics of celiac disease patients enrolled into the study.

	Patients *n* = 340, *n* (%) or Mean (SD)
Sex (Female) (%)	223 (65.6)
Age at diagnosis (Months)	76.6 (44.3)
Body mass index (BMI) percentile at diagnosis	38.1 (30.4)
Human Leukocyte Antigen (HLA)	
DQ2	291 (85.6)
DQ8	20 (5.9)
DQ2/DQ8	29 (8.5)
IgA Anti-tTG antibodies (U/mL)	110.0 (118.8)
IgA deficit	6 (1.7)
EMA+	304 (86.1)
Esophagogastroduodenoscopy performed	295 (86.8)
Marsh Classification at biopsy	
2	51 (15)
3	244 (71.8)
Biopsy not performed	45 (13.2)
Family history of celiac disease	93 (27.4)

**Table 2:** Symptom pattern and associated diseases in symptomatic celiac disease patients (*n* = 295) at diagnosis, between two consecutive five-year enrollment periods.

	Total *n* (%) *n*. 295	2004–2008 *n* (%) *n*. 98	2009–2014 *n* (%) *n*. 197	*p*
**Gastrointestinal symptoms**				
Diarrhea	24 (8.1)	12 (12.2)	12 (6.1)	0.071
Constipation	18 (6.1)	3 (3.1)	15 (7.7)	0.122
Abdominal pain	105 (35.6)	29 (29.6)	76 (38.8)	0.121
Changes in bowel habits	10 (3.4)	3 (3.1)	7 (3.6)	0.820
Bloating	83 (28.1)	32 (32.7)	51 (26)	0.234
Nausea/Vomit	30 (10.2)	10 (10.2)	20 (10.2)	1
Lack of appetite	32 (10.8)	7 (7.1)	25 (12.8)	0.145
Gastro-esophageal reflux	4 (1.4)	2 (2)	2 (1)	0.476
**Extra-intestinal symptoms**				
Hyposomia	48 (16.3)	21 (21.4)	27 (13.8)	0.094
Anemia	55 (18.6)	18 (18.4)	37 (18.9)	0.916
Slow growth	54 (18.3)	22 (22.5)	32 (16.3)	0.201
Headache	20 (6.8)	4 (4.1)	16 (8.2)	0.190
Epilepsy	4 (1.4)	1 (1)	3 (1.5)	0.722
Asthenia	25 (8.5)	3 (3.1)	22 (11.2)	0.018
Atopic dermatitis	29 (9.8)	14 (14.3)	15 (7.7)	0.072
Dermatitis herpetiformis	18 (6.1)	7 (7.1)	11 (5.6)	0.606
High transaminase levels	16 (5.4)	4 (4.1)	12 (6.1)	0.467
Muscle hypotrofia	4 (1.4)	3 (3.1)	1 (0.5)	0.075
Arthritis	3 (1)	0	3 (1.5)	0.218
Aphtosis	8 (2.7)	1 (1)	7 (3.6)	0.205
Recurrent infections	32 (10.8)	11 (11.2)	21 (10.7)	0.895
Tooth enamel alterations	1 (0.3)	0	1 (0.5)	0.479
**Associated diseases**				
Thiroiditis	2 (0.7)	2 (2)	0	0.045
Down’s syndrome	1 (0.3)	1 (1)	0	0.157
Allergies	40 (13.5)	18 (18.4)	22 (11.2)	0.092
Asthma	10 (3.4)	6 (6.1)	4 (2)	0.069

**Table 3:** Symptom’s pattern and associated diseases of symptomatic CD patients (*n* = 295) at different ages as follows: toddler age (0–3 years), primary school age (4–12 years) and high school age (>12 years).

	Toddler Age (0–3 Years) (*n* = 70) *n* (%)	Primary School Age (4–12 Years) (*n* = 200) *n* (%)	High School Age (>12 Years) (*n* = 25) *n* (%)	*p*
**Gastrointestinal symptoms**				
Diarrhea	10 (14.3)	13 (6.5)	1 (0.4)	0.091
Constipation	6 (8.6)	12 (6)	0	0.307
Abdominal pain	13 (18.6)	79 (39.5)	13 (52)	0.001
Changes in bowel habits	3 (4.3)	7 (3.5)	0	0.590
Bloating	40 (57.1)	38 (19)	5 (20)	<0.001
Nausea/Vomit	10 (14.3)	19 (9.5)	1 (4)	0.299
Lack of appetite	9 (12.9)	21 (10.5)	2 (8)	0.772
Gastro-esophageal reflux	1 (1.4)	3 (1.5)	0	0.827
**Extra-intestinal symptoms**				
Hyposomia	16 (22.9)	29 (14.5)	3 (12)	0.226
Anemia	12 (17.1)	39 (19.5)	4 (16)	0.845
Slow growth	21 (30)	30 (15)	3 (12)	0.015
Weight loss	5 (7.1)	2 (1)	0	0.011
Headache	0	14 (7)	6 (24)	<0.001
Epilepsy	1 (1.4)	3 (1.5)	0	0.827
Asthenia	0	19 (9.5)	6 (24)	0.001
Atopic dermatitis	3 (4.3)	21 (10.5)	5 (20)	0.066
Dermatitis herpetiformis	1 (1.4)	3 (1.5)	13 (52)	0.733
High transaminase levels	12 (17.1)	4 (2)	0	<0.001
Muscle hypotropia	1 (1.4)	3 (1.5)	0	0.827
Arthritis	1 (1.4)	2 (1)	0	0.830
Aphtosis	0	8 (4)	0	0.140
Recurrent infections	4 (5.7)	27 (13.5)	1 (4)	0.099
Tooth enamel alterations	0	1 (0.5)	0	0.787
**Associated diseases**				
Thyroiditis	0	2 (1)	0	0.618
Down’s syndrome	1 (1.4)	0	0	0.201
Diabetes type 1	1 (1.4)	2 (1)	1 (4)	0.457
Allergies	9 (12.9)	26 (13)	5 (20)	0.621

## Data Availability

The data presented in this study are available on request from the corresponding author. The data are not publicly available due to Ethics Committee policy.
